# Effect of Instrument Structure Alterations on Violin Performance

**DOI:** 10.3389/fpsyg.2018.02436

**Published:** 2018-12-06

**Authors:** Fabio Morreale, Jack Armitage, Andrew McPherson

**Affiliations:** Centre For Digital Music, School of Electronic Engineering and Computer Science, Queen Mary University of London, London, United Kingdom

**Keywords:** sensorimotor training, music cognition, expert performance, transparency effect, feedback and feedforward, generalization

## Abstract

Extensive training with a musical instrument results in the automatization of the bodily operations needed to manipulate the instrument: the performer no longer has to consciously think about the instrument while playing. The ability of the performer to automate operations on the instrument is due to sensorimotor mechanisms that can predict changes in the state of the body and the instrument in response to motor commands. But how strong are these mechanisms? To what extent can we alter the structure of the instrument before they disappear? We performed an exploratory study to understand whether and how sensorimotor predictions survive instrument modification. We asked seven professional violinists to perform repertoire pieces and sight-reading exercises on four different violins: their own, a cheap violin, a small violin, and a violin whose strings had been put on in reverse order. We performed a series of quantitative investigations on performance intonation and duration, and on bowing gestures and errors. The analysis revealed that participants struggled adapting to the altered instruments, suggesting that prediction mechanisms are a function of instrument configuration. In particular, the analysis of bowing errors, intonation, and of performance duration suggested that the performance with the reverse violin was much less fluent and precise than the performer's own instrument; the performance with the small violin was also sub-standard though to a lesser extent. We also observed that violinists were differently affected by instrument modifications, suggesting that the capability to adapt to a new instrument is highly personal.

## 1. Introduction

The relationship between performer and instrument is interesting both for its complexity and for the common experience “that the musical instrument has become part of the body” (Nijs et al., 2009). This phenomenon is explained by the theory of embodied music cognition (Leman, 2008; Loeffler et al., 2016), which suggests that the musical instrument is a *mediation technology* between the mind and a musical environment. At expert level, the instrument becomes transparent to the performer (Rabardel, 1995; Leman et al., 2010); the bodily operations of manipulating the instrument become automatic, so the performer's full attention can focus on the action of creating music (Nijs et al., 2009). In other words: the challenge of understanding performer-instrument interaction is precisely that the performer is not consciously thinking about the instrument while playing. Neuroscientists attribute this transparency to a number of internalized mental mechanisms that musicians acquire with time which guide complex motor activities needed to operate the instrument as if it was an extension of the body (Repp, 1999; Stambaugh, 2016).

This paper investigates the performer-instrument relationship from the point of view of its sensitivity to changes in instrument configuration, taking the violin as a case study. In contrast to the established approach of altering auditory feedback while keeping the physical interface the same (Repp, 1999; Pfordresher, 2005; Chen et al., 2008), we altered the instrument itself. We conducted an experiment in which violinists were asked to play exercises and repertoire on four violins with different physical characteristics: their own violin, an inexpensive student violin, a 1/4-size child's violin, and a full-size violin with the strings attached in reverse order. We observed and analyzed a number of factors, including intonation, fluency, bowing angles and bowing errors, describing their performance behaviors and the difficulties they faced. Elaborating on our results in the context of theories of motor learning, feedforward and feedback control and skill transfer, we reflect on the characteristics of sensorimotor prediction in expert music performance.

## 2. Background

### 2.1. Feedforward and Feedback in Musical Performance

The cognitive processes behind complex motor skills are a subject of intense ongoing research and debate (Engel et al., 2016). One stream of related work in neuroscience suggests that the mental representation that guides complex activities is composed of a combination of *feedback* and *feedforward* models (Wolpert et al., 1995; Zatorre et al., 2007; Ruiz et al., 2009; Yarrow et al., 2009; Stambaugh, 2016). The combination between the two models is described as a three-stage process by a motor control framework, called *optimal feedback control* (Todorov and Jordan, 2002). First, the feedforward model predicts changes in the state of a body part (or an object) in response to motor commands. Second, these predictions are combined with sensory feedback forming a judgment about the state of our body (or the object). Third, this judgment is used to adjust the gains of the sensorimotor feedback loop to maximize some measure of performance.

Applying this to the specific case of violin performance, the first stage allows the performer's brain to predict the sequence of movements necessary to perform the next musical material and detect errors before the sensory feedback arrives. In other words, the action-perception coupling in the violinist's brain forms predictions on the future state of her hands, which are compared with the state of her body and the consequences of her movements (Wolpert et al., 1995; Novembre and Keller, 2014). Such comparison allows the violinists to detect errors and consequently adjust the position of the hands (Wolpert et al., 1995; Novembre and Keller, 2014). In the second stage, the auditory and tactile feedback arrives and is processed, and in the last stage, the performer implements appropriately timed motor adjustments.

Some degree of feedforward control is imperative in music performance and other activities which unfold too rapidly to rely exclusively on sensory feedback with its inherent delay (Flanagan and Wing, 1997; Wolpert et al., 2001; Wagner and Smith, 2008). The internal feedforward model forms mental images of the intended sound before the sound is actually generated, updated in real time with the state of the body (Novembre and Keller, 2014). Evidence that pre-sensory adjustments to expected responses of movements is happening at a neural level comes from a study by Maidhof et al. (2009) which found that expert piano players played wrong notes with less motor vigor (velocity) than accurate notes, and that EEG recordings showed differences between accurate and wrong notes prior to keys being pressed.

**Figure 1:**
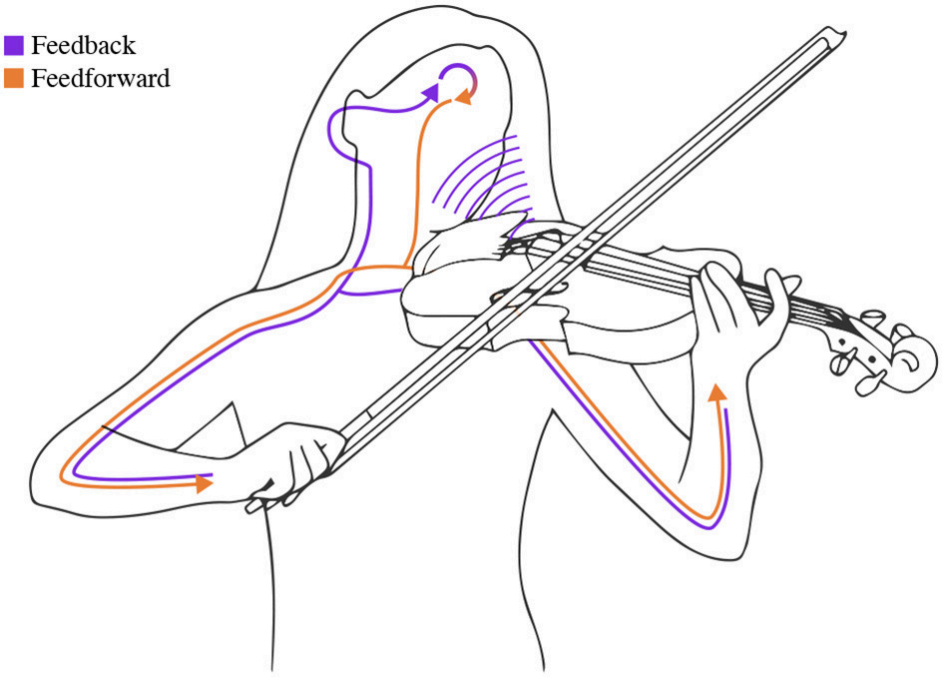
When a musician performs, feedforward and feedback mechanisms interact. The motor system controls the fine movements needed to produce the sound (feedforward). The sound is then processed by the auditory system (feedback), which uses this information to adjust motor control.

The role of feedforward control was also suggested by Lashley (1951), who found that the processing of auditory feedback is too slow to manage error correction for the fast motor sequences of piano performance at a high tempo. As a consequence, Repp (1999), Yarrow et al. (2009), and Mazzoni and Krakauer (2006) agree that the ability to accurately and expressively perform a piece of music is strictly dependent on the interaction between feedforward and feedback models (Figure 1), which can be acquired and updated via motor learning, i.e., “the acquisition of forward and inverse internal models appropriate for different tasks and environments” (Wolpert et al., 2001). Of course, in musical performance, instrumental gestures can also serve as communication with an ensemble or audience in addition to their function in producing sound (Biasutti et al., 2016). Different mental processes may be involved in this communication, which are beyond the scope of this paper.

A common method of investigating motor learning for musical performance is to alter or suppress auditory feedback and observe the extent to which the performance is disrupted. Repp (1999) performed an experimental study with pianists in which they investigated whether performance deteriorates when the auditory feedback is absent. Their result suggests that it does not: musicians seem to rely on an internal representation of the music to guide and pace their performance, even in the absence of any audible sound. The authors also suggest that such sensory deprivation does not impact on the expressive timing and dynamics of music performance. Only pedal use was seriously affected by sensory deprivation, perhaps because pedaling depends on auditory feedback to a greater extent. Rather, it is the fine-tuning of a performance that depends on sensory feedback to a greater extent (Repp, 1999). Chen et al. (2008) found that during hand position shifts on the cello, finger position accuracy suffered in the absence of auditory feedback. This observation also resonates with the conclusion drawn by Rosenbaum (2009), who argued that the subtleties of interaction of skilled motor activities are aspects that seem to suffer the most the absence of feedback.

A similar approach based on disruption of auditory feedback was proposed by Ruiz et al. (2009), who investigated the feedforward mechanisms acquired by musicians through extensive training. They conducted an experimental study in which pianists retrieved memorized pieces at a fast tempo in the presence or absence of auditory feedback. The objective was to investigate the predictive (feedforward) error detection during piano performance. Their results are aligned with previous research (Bernstein, 1966; Wolpert et al., 1995) stating that the performance of trained motor actions requires the action-monitoring system to be perfectly tuned to be able to predict in advance potential errors.

Other experimental studies have investigated the effect of altering, but not suppressing, auditory feedback: when the auditory feedback is present but altered, the disruptive effect seems to increase. In a study analysing auditory delays in speech, Vaxes (1963) reported that delaying auditory feedback results in partial disruption of the abilities to speak correctly. Among other articulatory changes, vowel length is prolonged and consonants tend to be repeated. In the musical domain, Pfordresher (2005) explored whether mismatches between auditory feedback and planned outcomes influenced fluency in piano performance. The results suggested that altering pitch contents result in performance disruption only when the sequence of altered pitches is structurally similar to the planned sequence. By contrast, disruption of performance was not observed when the feedback was absent and when the pitches were randomly selected. The authors hypothesize that serial shifts disrupt planning-related, memory-based processes rather than execution-based processes.

Pfordresher and Palmer (2006) conducted a further experiment investigating the effect of alterations of auditory feedback on piano performance. The feedback was altered by presenting pitches of other sequence events, specifically pitches intended for the past (*delay*) or the future (*prelay*). The results suggest that all alterations resulted in more pitch errors when the altered feedback proposed proximal and metrically similar events. Patterns of serial-ordering errors suggested that performers compensate for the disruptive effects of altered feedback by changing event activations during planning. Interestingly, delays and prelays increased the tendency to play pitches in the direction opposite to the feedback, suggesting that performers seem to alter planning to compensate for altered feedback. Analysing this result from the lenses of the *optimal feedback control* framework (Todorov and Jordan, 2002), such compensations resulted in adaptation of the feedforward model that comes into play at the initial phase of the motor control.

Another experimental study that made use of auditory disruption to test musicians' perception was conducted by Hafke-Dys et al. (2016). The authors aimed to assess musicians' motor reactions to frequency perturbations. Modifications of the fundamental frequency were introduced in violin performance and the reaction of the violinists to these modifications was computed. Their results suggest that musicians' compensations were precise, and that the degree of precision was independent of the value of frequency modification.

In their review of music-related behavioral and neuroimaging research, Novembre and Keller (2014) concluded that the perception and action are strongly coupled in musicians as a result of learning a specific sensorimotor task. The authors suggest that a possible reason for this ability of the brain to “represent a perceived action in terms of the neural resources necessary for producing it.” This coupling seems to be responsible for generating predictions about one's own actions and the actions of others, and it might be assessed by designing experimental tasks requiring the production of complex musical sequences in real or virtual interaction settings.

Extensive musical training also results in differences in music perception. The differences between expert and novice listeners are widely discussed in the literature: musical training results in different perception of musical style (Meyer, 1967), communicated emotions (Bigand et al., 2005; Morreale et al., 2013), and which sonic aspects a listener focuses on: novices tend to focus more on secondary parameters - louder vs. softer, higher vs. lower - rather than on melody and harmony (Gromko, 1993).

### 2.2. Skill Transfer and Interference

Skilled motor behaviors can be adapted to different contexts thanks to the ability of humans to apply what has been learned in one context to another context. This capability of transferring past experiences onto new ones is called *generalization*. Generalization is termed *transfer* when it is beneficial and *interference* when it is detrimental (Krakauer et al., 2006). Similarly to the presence of feedback and feedforward mechanisms, both types of generalizations are dependent on the history of training (Krakauer et al., 2006). Generalization has provided researchers with an important tool to understand the specificity of learning. In particular, new understanding of the changes that have occurred during learning is often gained in experimental studies by testing whether the effects of training extend to untrained movements and novel contexts (Poggio and Bizzi, 2004). While applying these sensorimotor adaptive mechanisms, the brain is not just concentrating on the new information in the environment, it is also suppressing the automatic responses of existing pathways (Gentili et al., 2015).

A review by Bock (2013) suggests that adaptation is based on a number of common mechanisms subdivided into different modules, each specialized for specific functions, particularly rotation, axis-inversion, and scaling. The accuracy of task-performances when rotation is altered depend on the magnitude of rotation with respect to the trained one. Axis-inversion seems to be direction-selective, i.e., it only operates for a limited range of movement directions around the practiced direction. By contrast, scaling modules seem to be less direction-selective (Bock, 1992; Krakauer et al., 2000). These theories are usually tested in experimental design in which the visual feedback is perturbed forcing a translation or a rotation of the original signal (Shadmehr and Mussa-Ivaldi, 1994; Taylor and Ivry, 2013; Telgen et al., 2014). These perturbations create differences between expected and actual visual feedback which are used to update the internal model (Taylor and Ivry, 2013).

Generalization *transfer* was observed when movements differ in amplitude and duration (Goodbody and Wolpert, 1998), when they involve the same joint rotations (Shadmehr and Mussa-Ivaldi, 1994), and when a translation of the actual workspace is performed (Ghahramani et al., 1996). By contrast, different direction of movements and visuomotor rotation seems to *interfere* with the capability to generalize learning (Krakauer et al., 2000; Malfait et al., 2005). (Telgen et al., 2014) investigated generalization of movements when the new conditions operated an axis-inversion mapping of visual feedforward (left-right reversal over a mid-sagittal axis), finding that the reversed mapping had a similar error rate to the original but a significantly longer reaction time. Interestingly, in the reverse case, corrective responses were initially made in the wrong direction and reoriented only in the late phase of the response. The authors conclude that feedforward and feedback control both require additional processing time in the beginning of learning and then are increasingly automatized. Laeng and Park (1999) tested performance accuracy on a keyboard with the high-to-low axis reversed, finding an interference effect whereby previous keyboard-playing experience led to greater error rate in the reverse condition compared to novices playing the same instrument.

The level of sensorimotor similarity between old and new experiences is also an important aspect for generalization. For example, previous expertise in typing on a QWERTY keyboard interferes with the use of an AZERTY keyboard, while learning to type with a large QWERTY keyboard may transfer to typing on a smaller QWERTY keyboard (Bérard and Rochet-Capellan, 2015).

It is worth noting that most of the experiments supporting theories of feedforward and inverse models have involved relatively simple movements (pointing to a target, rotating a handle) with few degrees of freedom. The same observation applies to most studies on skill transfer and interference reported in this section. It is an open question as to how well theories derived from learning of simple or restricted movements generalize to more complex skills like violin performance (Wulf and Shea, 2002).

## 3. Objectives

The preceding review indicates that expert musical performance relies heavily on automated motor programs trained over an extended time. The robustness of these motor programs was demonstrated by the ability of performers to cope with absence of, and certain alterations to, auditory feedback. With limited exceptions (Laeng and Park, 1999), there have been few studies of the ability of performers to adjust to changes in the physical structure of their instruments that require different motor actions to achieve the same familiar sounds.

In this paper, we seek to study the alteration of feedforward pathways: the transfer function between physical action and resulting sound. This transfer function is largely a function of the mechanical construction of the instrument, although its construction also changes what actions are suggested to the performer (Jack et al., 2017; Tuuri et al., 2017), such that the term “instrument” effectively signifies not only a mechanical object but a set of performance practices. For this section, however, we focus on the narrower point that changing the mechanical construction of the instrument alters its action-sound relationships and thereby potentially disrupts established sensorimotor learning that depends on those specific relationships. We are particularly interested in the effect of instrument design on the subjective experience of *transparency*, wherein after years of practice an instrument recedes from conscious attention while in use (Nijs et al., 2009). Rabardel (1995) describes this type of transparency as *functional*. In contrast to *relational* transparency, which refers to the (non-)interference of the musical instrument with the direct perception of the musical environment, *functional* transparency exists when musician can respond to the musical environment without conscious reflection.

The concept of instrument and technology transparency in human activities is thoroughly discussed by Dourish (2001) when presenting his theory of Embodied Interaction. Dourish's work is inspired by pheonomenology (a philosophical school of thought concerned with how humans perceive and act in the world) and in particular by Heiddeger's concept of tools of *present-at-hand* and *ready-to-hand*. Dourish's classical example to explain this concept is the computer mouse:

Consider the mouse connected to my computer. Much of the time, I act through the mouse; the mouse is an extension of my hand as I select objects, operate menus, and so forth. The mouse is, in Heidegger's terms, *ready-to-hand*. Sometimes, however, such as when I reach the edge of the mousepad and cannot move the mouse further, my orientation toward the mouse changes. Now, I become conscious of the mouse mediating my action, precisely because of the fact that it has been interrupted. The mouse becomes the object of attention as I pick it up and move it back to the center of the mousepad. When I act on the mouse in this way, being mindful of it as an object of my activity, the mouse is *present-at-hand*.

An unanswered question is whether there exists a *transparency bandwidth* for musical instruments, i.e., an amount by which an instrument can be altered before its transparency to the performer breaks down and the instrument goes back to a present-at-hand mode.

We present an exploratory study that perturbs established perception-action couplings and examines the effects on performance accuracy and performer experience. Seven violinists were each asked to play four violins of varying familiarity and physical configuration, some specifically chosen to provoke disruption of existing sensorimotor programs. In comparison to studies of expert vs. novice performers (MacRitchie, 2015), experts playing on unfamiliar instruments retain a highly developed sense of auditory perception but lack sensorimotor skills fully adapted to the instrument. We limit our study to the motor control involved in technical aspects of playing, considering exclusively *sound-producing gestures* in the taxonomy by Jensenius and Wanderley (2010), without seeking to assess effects on musical expression. This study therefore offers insight into the relative roles of physical familiarity and general musicianship in expert performance, and it examines the robustness of existing sensorimotor skills to changes in physical form.

## 4. Methods

### 4.1. Participants

We recruited expert violinists via an open call distributed through our research network and local conservatoires. Participants were requested to be one of: professionally active violinists, currently enrolled in a conservatoire, or recently graduated from conservatoire. Seven violinists (5 female) answered and took part in the study. The participants had been playing violin from 14 to 38 years (average 24.4). Five of them are professional violinist (P2-P7) while P1 regularly performs but is not professionally active^1^. Performers were paid £75 for their participation^2^. The study was approved by the ethics board at Queen Mary University of London.

### 4.2. Setup

**Figure 2:**
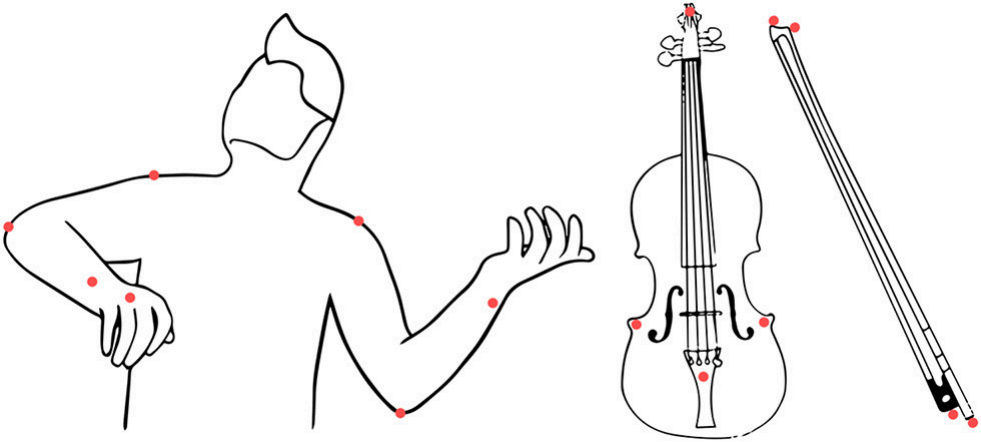
Placement of the markers on the violinist body, the violin, and the bow.

The study took place at Queen Mary University of London, in a black-box performance space equipped with a Vicon motion tracking system consisting of 12 infrared cameras. We used the motion capture system to track movement of the performer and the bow-instrument interaction. To do so, a number of markers were attached to the bow, violin, and the performer's body as shown in Figure 2. Performances were also video recorded from two camera angles and audio recorded with a stereo microphone several feet from the violin. Each study session was allocated 3 h, including two breaks of 15 min each, though most sessions took between 2 and 3 h total.

### 4.3. Instruments

Each violinist performed on four violins. The violin was chosen as the instrument of study not only for its popularity and the corresponding ease of recruiting participants, but also because it is relatively easily modified to simulate some common sensorimotor transformations (i.e., scaling and axis-inversion) (Bock, 2013).

The presentation order of the instruments was the same for all participants. The first violin was the performer's own instrument (labeled *personal* in subsequent analyses). The second violin was a factory-made student violin by the company Stentor (labeled *cheap* in subsequent analyses). Though it was otherwise an ordinary unaltered instrument, it was selected to test the player's sensitivity to the particular instrument they most frequently play, as compared to a generic instrument of similar proportions. The third violin (*small*) was a 1/4-size violin, typically designed for children aged 4–6, whose size is approximately 77% of a full-size violin. It was chosen to test sensitivity of violin technique to physical scaling. The fourth violin (*reverse*) was a full-size Stentor student violin whose strings had been put on the opposite order, where the leftmost string is the high E, followed by A, D and finally low G as the rightmost string.

During the pilot study, we discussed with P0 whether the reverse violin should have the bridge reversed in direction as well. (Violin bridges are nearly but not entirely symmetrical, with the high E typically elevated less than the low G.) We ultimately decided to leave the bridge in its normal orientation since this is more ergonomic to play and retains the same set of four bow angles as a conventional instrument, but in reverse order. The violinists were asked to use their own bow for the first condition (*personal*). For the remaining three conditions, they were given a standard student-quality bow. While children playing a 1/4-size violin typically also use a smaller bow, we chose to retain the full-size bow across conditions to focus on the effects of scaling the instrument size.

### 4.4. Procedure

**Figure 3:**
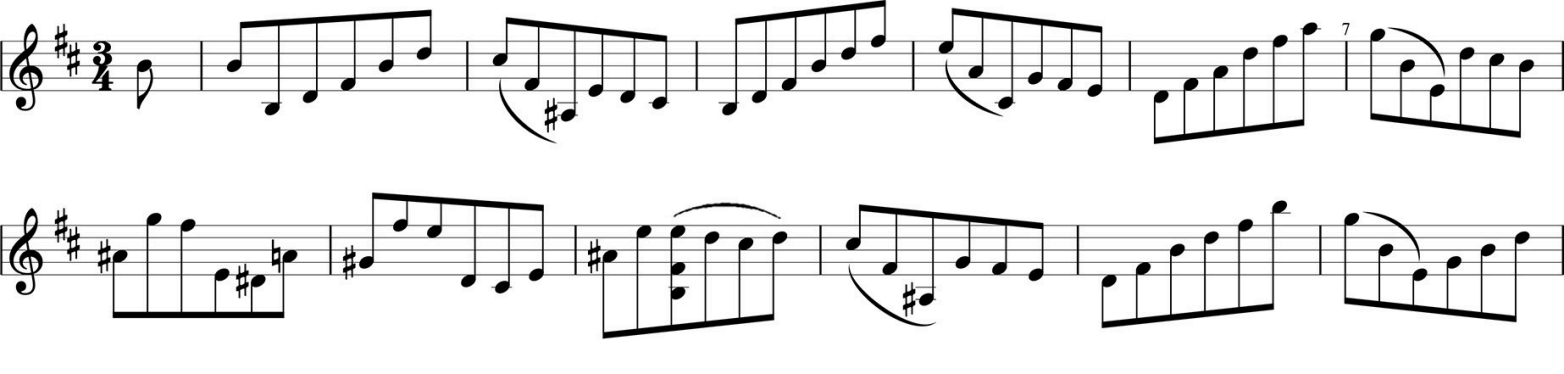
The first two staves of the Bach *Courante* from BWV 1002 that all performers played in each condition.

Before starting, the violinist received an information sheet stating the objectives of the research and that the experiment was going to be videotaped for research purposes. After they read the Information Sheet, they were then asked to sign a consent form. After that, one of the researchers attached reflective markers to the performer's body and his/her personal bow and violin (the other violins and bow were pre-fitted with markers). After a few minutes of warming up, the violinist was invited to perform a series of exercises and pieces, which appeared in the same order for each participant, repeated for each of four violins (we did not give performers any instruction on the tempo):

1. G major scale, 3 octaves ascending and descending, 1 note to the bow.
2. Another major scale, chosen from A, B♭, C, or D major. 3 octaves ascending and descending, 1 note to the bow.
3. Two arpeggios, one minor, one major, chosen from G, A, B♭, C, and D. 3 octaves ascending and descending, 2 notes to the bow.
4. *Courante* from the Bach Violin Partita no. 1 in B minor, BWV 1002, edition Breitkopf und Härtel 1879 (Figure 3), played without repeats. An electronic score of this piece was sent to each violinist 2 weeks before the session to give them time to familiarize themselves with the piece.
5. Three excerpts of the player's choice from their own repertoire, which might include solo, chamber or orchestral parts. The player was asked to play all three on their own violin, and for subsequent violins, we chose two of the excerpts (the same each time).
6. Three sight-reading exercises at increasing difficulty, taken from *ABRSM Violin Specimen Sight-Reading Tests*, 2012 edition. The three excerpts were from ABRSM grades 4, 6, and 8, respectively. Different excerpts were chosen for each violin and for each participant.

The order of presentation of violins was *personal, cheap, small, reverse*, following an order of instruments which we expected would go from most to least familiar. Participants were not informed in advance about the nature of the violins they would be playing. Violins were hidden from view before presenting them to the participant. No specific guidance was provided on tempo or expressive qualities of each performance.

Following the completion of the above exercises on each violin, participants were interviewed by two researchers. The interviews were arranged as semi-structured, narrative conversations. In this interview format the questions are designed to provoke narration from the interviewee, while the researcher can adapt the flow of topics and, if need be, encourage follow-up questions (Bernard, 2000).

As a final exercise, some participants were asked to play the Bach *Courante* on the reversed instrument as if the strings were in their standard order, ignoring the sounding pitches^3^.

### 4.5. Hypotheses

After years of playing a particular instrument, players become attuned to its subtlest details and idiosyncrasies such that changing to any new instrument requires a period of re-adjustment (Bijsterveld and Schulp, 2004). From the perspective of action-perception coupling (Novembre and Keller, 2014), we suggest this re-adjustment period might be explained as a process of updating the forward and inverse internal models (Wolpert et al., 2001). However, we expect the three unfamiliar violins (*cheap, small, reverse*) to elicit different reactions and experiences, which would manifest in magnitude and types of error, fluency of performance, and self-reported experiences of the musician.

The *cheap* violin is included in the study primarily to assess the effects of changing from the musician's own violin to *any* other violin. We might expect to see subtle reductions in performance accuracy, depending on the factors that are measured, alongside a potentially large reduction in self-reported quality of the playing experience.

We expect larger observable differences in performance from the scaled (*small*) and axis-inversion (*reverse*) conditions. If the mental process of musical instrument learning creates shared representations between the expected sound of an action and the motor operations needed to produce it, then that recall will be disrupted when the instrument is changed. Performers will no longer be able to rapidly draw on that shared representation, leading to increased attention requirements.

On the *small* instrument, we hypothesize that the performer's feedforward mechanisms would lead to inaccurate left-hand finger placement producing pitches that are systematically too high, without fundamentally affecting the spatial organization of the pitch space, as the performer's natural finger spacings for a given location on the fingerboard would be too wide for the small instrument. If the difference between expected and actual pitch is small enough, the performer could apply their normal means of pitch correction through auditory feedback (Chen et al., 2008), which might manifest in notes that begin with a large pitch error which reduces over time. In terms of bow technique, we might observe inadvertent double-stops or poor tone quality caused by the smaller gap between strings or the different response of the shorter, low-tension strings on the small instrument. Nonetheless, if the performer can rely on established motor programs (however inaccurate) for both initial actions and corrective adjustments, we would expect the performance on the small violin to remain essentially fluent.

We expect the *reverse* instrument to show the greatest disruption. Though the scaling of the fingerboard is the same as an ordinary violin, the reverse order of the strings means that the performer's feedforward mechanisms will cause the wrong string to be played. Moreover, this type of error might not be corrected through familiar feedback mechanisms: it is not resolvable by moving the left-hand finger placement, and the first reaction to bowing the wrong string might be to move further in the wrong direction, further emphasizing the errors. The performer may have to use conscious attention rather than established internal models to produce the correct outcome, leading to a performance which is not fluent and a complete saturation of attention which prevents focus on higher-level nuances of performance.

To investigate these points, we considered several quantitative metrics of each performance: intonation accuracy, duration of performance, bow angle accuracy, and number and type of bowing errors. The first of these was measured during the initial G major scales, while the others were measured during the Bach *Courante*^4^. We also elaborate on the comments collected from the violinists in the post-study interviews.

## 5. Intonation Accuracy

Previous work (Chen et al., 2008; Hafke-Dys et al., 2016) suggests that string players can guide their initial left-hand finger placements in the absence of auditory feedback, but that they use auditory feedback to correct errors in intonation. In the following analysis, we seek to observe the performer's first performed pitch at the beginning of each note, before sensory feedback is processed, in comparison to the pitch at the end of each note. Our hypotheses would suggest that initial intonation will be systematically too high on the *small* violin, but that no significant differences should exist between the other three violins, since they will have nearly the same string length.

**Figure 4:**
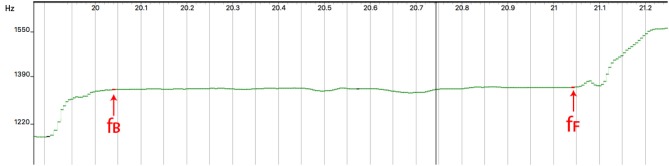
The output of the pYIN analysis on a window of around 1 s. *f*_*B*_ indicates the beginning of the note, *f*_*F*_ indicates the end of the note as manually computed.

For this section, we analyse a 3-octave G major scale played by each player on each instrument. The audio recordings were analyzed with Sonic Visualiser^5^ using *pYin* (Mauch and Dixon, 2014), a monophonic pitch tracking algorithm. Robust note onset detection on string instruments remains a challenging problem, so notes were segmented manually by integrating a visual analysis of the spectrograms with the spreadsheet of the frequencies generated by pYin. To reduce the risk of being unsystematic, a series of strategies were adopted. For each note *n*, the beginning of the note *f*_*B*_(*n*) was associated with the first flat part (Figure 4) of the signal after note transition. The end of the note *f*_*F*_(*n*) was associated to the instant before the signal artifacts that occur when a performer gets ready to change note (Figure 4). We also computed *f*_*A*_(*n*), the mean frequency of all samples between *f*_*B*_(*n*) and *f*_*F*_(*n*). We then calculated the average beginning *m*_*B*_(*n*) and ending *m*_*F*_(*n*) part of the note by averaging a window of 20 frames (approximately 100ms). *m*_*B*_(*n*) was computed using a window that comprised *f*_*B*_(*n*) and the successive 19 frames. The end note, *m*_*F*_(*n*), was computed using a window that comprised the *f*_*F*_(*n*) frame and the 19 preceding frames.

For each note *n* of the scale we calculated the expected frequency *f*_*E*_(*n*) as

(1) $$\begin{array}{l} f_{E}\left(n\right)=m_{0}2^{d\left(n\right)/12} \end{array}$$

where *m*_0_ is the average frequency of the first note (the lowest G), and *d*(*n*) is the difference in semitones between the note *n* and the lowest G. We then calculated the accuracy of the performances at different moments by calculating, for each note, the error in cents^6^ for the beginning (*a*_*B*_(*n*)) and the end of the note (*a*_*F*_(*n*)) and for the average frequency *a*_*A*_(*n*)

(2) $$\begin{array}{l} a_{B}\left(n\right)=1200\text{lo}{\text{g}}_{2}\left(m_{B}\left(n\right)/f_{E}\left(n\right)\right) \end{array}$$

(3) $$\begin{array}{l} a_{F}\left(n\right)=1200\text{lo}{\text{g}}_{2}\left(m_{E}\left(n\right)/f_{E}\left(n\right)\right) \end{array}$$

(4) $$\begin{array}{l} a_{A}\left(n\right)=1200\text{lo}{\text{g}}_{2}\left(m_{A}\left(n\right)/f_{E}\left(n\right)\right) \end{array}$$

It has to be noticed that this specific analysis was performed to understand intonation issues rather than detecting blatant mistakes that were expected with the reverse violin. Thus, all the *wrong notes*^7^ were excluded for the examination discussed in the next section.

### 5.1. Results

**Figure 5:**
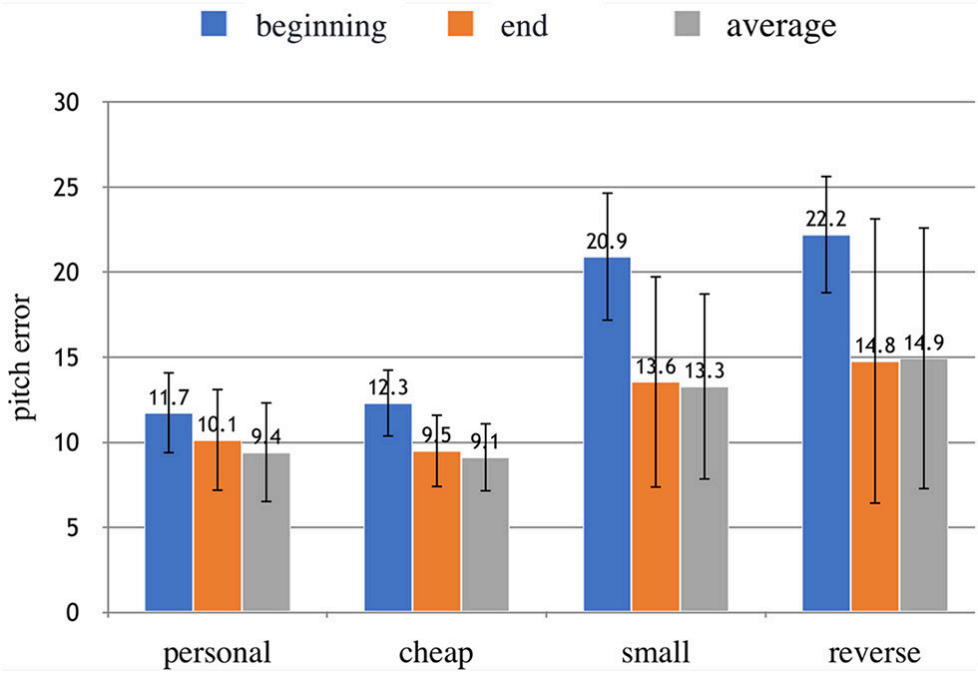
Absolute values of the pitch error in the different conditions: at the beginning and at the end of each note, and their average value. The error bars show the StD. A high value indicates a high pitch error, thus a bad accuracy.

The values of the means and the StD of the absolute values of pitch errors with the four instruments are shown in Figure 5. The three columns refer to the three stages of the note: beginning (blue), end (orange), and average (gray). The figure suggests that performers were less precise with the altered violins, and in particular during the initial transient of the note. The initial accuracy with both altered instruments is considerably higher than the normal conditions. This seems to disprove our hypothesis that only scale alteration would have disrupted the ability to predict the note position.

The analysis of the StD (error bars) offers other important insights. The StDs of *a*_*B*_ are much less than the StDs of *a*_*F*_ and *a*_*A*_, suggesting that all performers struggled to predict the initial position of the notes but then they had different capabilities to adapt to these instruments.

The statistical significance of these observations was tested. First, three one-way within-subjects ANOVAs were conducted on the absolute values of the pitch errors in the three moments (beginning *a*_*B*_, end *a*_*F*_, and average *a*_*A*_). There was a statistically significant effect of the type of instrument on the accuracy at the beginning of the notes *a*_*B*_, accounting for a large proportion of the variance: *F*_(3, 18)_ = 27.371, *p* < 0.00025, partial μ^2^ = 0.82. A significant difference was also found for the average note accuracies, but it accounted for a lower proportion of the variance: *F*_(3, 18)_ = 3.227, *p* < 0.05, partial μ^2^ = 0.35. Finally, the influence of the instrument on accuracy at the end of the notes was not statistically significant.

A paired *t*-test (one-tailed) was performed to compare the begin vs. final cents in all conditions (absolute values). We performed a one-tailed test as we hypothesized the pitch error to be higher at the beginning of the notes. As expected, the pitch error at the beginning *a*_*B*_ was statistically higher than the pitch error at the end of each note *a*_*F*_ for all the instruments, confirming the fine-tuning effect of the feedback mechanism. The magnitude of the differences in the means *d* was large in all cases (small: *d* = 1.46, cheap: *d* = 1.36, reverse: *d* = 1.25, personal: *d* = 0.58).

**Figure 6:**
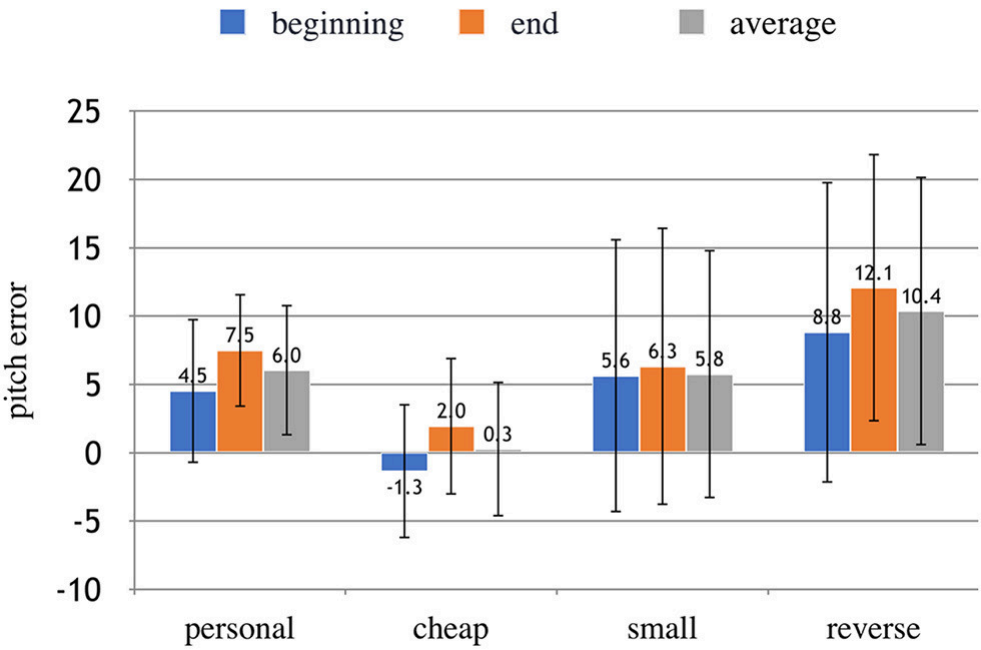
Non-absolute values of the pitch error in the different conditions at the beginning and at the end of each note. The error bars show the StD. A high value above zero means that the note is particularly sharp.

The non-absolute values of accuracy were also computed to explore whether different conditions were systematically sharp or flat relative to the open G string. We expected the small instrument to show a systematically sharp (too high) finger placement for the initial intonation given its smaller scale. The results, shown in Figure 6 suggests that the notes played were generally sharp. A one-way within-subjects ANOVA was conducted on the non-absolute values of *a*_*A*_, *a*_*B*_, and *a*_*F*_. There was not a statistically significant effect of the type of instrument on the direction of the error.

**Figure 7:**
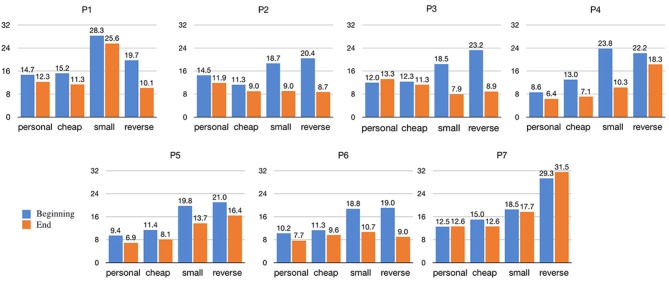
Means of absolute pitch errors for each performer at the beginning and at the end of the notes.

We further inspected the apparent difference among performers, which caused high values of StD in the altered instruments (especially in the final and average part of the note). For each player, a 4 (instrument) x 2 (moment: beginning vs. end) repeated-measures analysis was conducted on pitch error measured in cents for each note of the scale. As expected, for all performers the intonation of the final part of the note was significantly better than the initial phase of the note. However, the main effect of instrument type was statistically significant only for P1, P4, P5, P6, and P7. The accuracy of P2 and P3 did not significantly change among different instruments. Figure 7 shows for each performer the means of the pitch error for all the instruments at the beginning and at the end of the notes. It can be observed that performers had very different experiences with error correction. P2, P3, and P6 managed to correct their intonation after the feedback arrived even with the altered instruments. P1 did not correct the error with the small violin, whereas P1 and P7 only struggled with the reverse violin. P5 struggled with both altered violins, and in particular with the reverse one. Oddly, the observed intonation accuracy of P3 at the beginning of the note was better than that at the end of the note for the personal violin. The footage of the performances explained the reasons for this behavior: when performing the scales on the non-altered instruments, P3 made substantial use of vibrato thus the final frequency of the note *a*_*F*_ was often displaced with respect to the central frequency.

### 5.2. Discussion

These results indicate that violinists' initial finger placement for both altered instruments was less accurate than the non-altered violins, suggesting that existing sensorimotor predictions are not malleable enough to be able to compensate for the difference in scale and string orientation before the auditory feedback arrives. This observation supports our hypotheses in the case of the small violin: the initial pitch error was higher than the non-altered conditions. However, the initial pitch error was higher also for the reverse violin, a result that contrasts with our hypothesis and that does not have an obvious explanation. One possibility is that the positioning of the left hand means that the fingers extend diagonally across the strings, such that inverting the order of the strings leads to inaccurate expected positions of each note.

Another notable result is that violinists (at least some of them) were able to quickly correct the intonation even with the altered instruments (the influence of the instrument of the accuracy at the end of the notes was not significant). This fact partially challenges our hypothesis that violinists would not be able to use their familiar feedback mechanisms to correct initial predictions under axis-inversion conditions, though it is notable that reversing the order of the strings nonetheless retains the usual low-to-high pitch relationship on each individual string. With respect to the direction of the error, we expected that performances on the small violin would be systematically sharp given its smaller size relative to the performer's hand. The results suggest that this was indeed the case; however, the behavior of the small violin in this respect is statistically similar to the other violins.

The analysis also showed that, once the feedback arrives, violinists were able to correct their positions, confirming that presence of a predictive mechanism that is then updated when the sensory feedback arrives. In all conditions, even with violinists' own instrument, the mean intonation error was higher at the beginning of the notes than at the end. Although Figure 5 shows that the pitch errors with the altered violins seem to be higher than those of the non-altered violins, the main effect of type of instrument on the pitch error was not significant, probably due to the high variance.

## 6. Performance Duration

We measured the duration of each performance of the Bach *Courante* excerpt, mm. 1-32 (up through the first repeat). We expected instrument alterations to reduce the ability to execute the correct physical movements through automated internal models, thus performers would need to deploy conscious attention to their movements. This, in turn, could necessitate a slower or even halting performance, similar to a performer who is not an expert sight-reader encountering an unfamiliar piece. Table 1 shows how long it took each violinist to play the Bach *Courante* (first 32 bars) on each violin. (The performers were not given any instruction on the tempo).

**Table 1:** Duration, in seconds, of each performance of the Bach *Courante* from BWV 1002, mm. 1-32.

	**Personal**	**Cheap**	**Small**	**Reverse**
P1	66	69 (104.5%)	59 (89.3%)	172 (260.6%)
P2	47	48 (102.1%)	46 (97.8%)	94 (200.0%)
P3	46	48 (104.3%)	46 (100.0%)	110 (239.1%)
P4	56	55 (98.2%)	54 (96.4%)	117 (208.9%)
P5	49	51 (104.0%)	49 (100.0%)	237 (483.6%)
P6	50	53 (106.0%)	58 (116.0%)	120 (240.0%)
P7	47	46 (97.8%)	44 (93.6%)	127 (270.2%)
Mean	51.57	52.85	50.85	139.57
Std	2.71	2.93	2.31	18.61

*The percentages refer to the time of the performance in relation that of the personal violin*.

The table shows that performances on the reverse violin were substantially longer than any of the other three violins. A paired *t*-test (one-tailed: we hypothesized performances with the altered instruments to be longer) between the personal violin and the other three conditions showed that the difference was statistically significant only for the pair *personal-reverse* (*t* = −4.873, *p* < 0.0025). The mechanism behind these longer durations was not simply a slow, steady tempo: rather, all performers on the reverse violin produced halting performances and a significant number of bowing errors, explored in detail in a following section. This effect was not observed with any of the other violins.

### 6.1. Discussion

The results support our hypothesis that axis-inversion requires performers to use conscious attention rather than established internal models to perform a piece thus resulting in a non-fluent performance. It is also notable that the small violin, despite its unfamiliarity, shows hardly any change in performance duration compared to the personal violin. The reverse violin shows larger variability in duration between performers compared to any of the other violins, confirming the observation that the capability of sensorimotor skills to adapt is highly personal. For example, P5 took 2.5 times longer to play the passage than P2 on the reverse violin (237 vs. 94 s), while their performances on their own violins are of similar length (49 vs. 47 s). Together, these results suggest that amongst the tested conditions, axis inversion has a uniquely disruptive effect on the automatic responses of the musician.

Another notable result comes from the analysis of the duration of the Bach Courante played on the reversed instrument as if the strings were in their standard order (i.e., not considering the actual pitches that are produced). All the four violinists that were asked to play this condition (see footnote 3 in Section 4) performed much more fluently than in the preceding reverse condition, with significantly shorter performance durations: P4, P5, P6, and P7, respectively, took 56 (100%), 54 (110%), 66 (132%), and 48 (102%) s.

## 7. Bowing Errors

We observed a set of bowing errors that were unique to the *reverse* violin and not observed on any of the other three instruments. The errors were similar in nature across all seven participants. We performed an analysis of the audio and video of the Bach *Courante* performances to categorize the errors made on this instrument. Leaving aside problems of intonation, tone quality, and tempo that might be found occasionally in any performance, we identified four types of errors that occurred only on the reverse violin:

- **Wrong string (WS)** errors, where the performer bows the wrong string, but with the left-hand finger placed in what would be the correct position on the correct string;^8^
- **Double stop (2S)** errors, where the performer bows two adjacent strings rather than one;
- **Open string (OS)** errors, where the performer bows a wrong string with no left-hand finger placed on that string;
- **Wrong finger (WF)** errors, where the performer bows the correct string but with an incorrect finger choice to play the indicated note. To distinguish from intonation problems, WF errors were only identified when the played note was at least 2 semitones away from the target.

Table 2 shows the number of each type of error each player committed over the 33 bars of the Bach Courante (198 notes). After playing a WS error, each player nearly always paused then attempted to play the same note again. In a few cases, players again made an error, requiring more than 2 tries to correctly play the note. In Table 2 the first number for each player counts only the first attempt (i.e., max one error per note) while the second number (after the plus) shows the number of additional incorrect tries.

**Table 2:** Number of errors on reverse violin in first 32 bars of Bach Courante.

	**WS**	**2S**	**OS**	**WF**
P1	35 (+1)	7	1	5
P2	2 (+0)	8	1	0
P3	9 (+3)	22	2	0
P4	16 (+0)	7	1	0
P5	34 (+12)	28	3	3
P6	13 (+7)	1	2	9
P7	20 (+1)	7	1	2
Total	129(+24)	80	11	19

*WS, wrong string; 2S, double stop; OS, (incorrect) open string; WF, wrong finger*.

Overall, WS is the most common type of error. We observed a median of 20 such errors in the excerpt. However, the table shows a wide disparity in performance across players, ranging from 2 to 35 errors. 2S errors, which involve hitting an inadvertent double stop, were the second most common type of error (median = 7), and also showed wide disparity across players (range 1–28). Though OS and WF errors were less common, WF errors also varied widely across players.

Table 3 shows a breakdown of the WS errors by strings. WS errors are much more common on the inner strings than the outer ones; the most common error, accounting for 56% of all WS errors, is to swap the D and A strings. Swapping these strings matches how the passage would be played if the violin were strung normally, however the effect of playing the mirrored string is relatively uncommon on the outer strings: only 5 times is the E string chosen when the G should have been played, and only 2 times does the reverse happen, together accounting for just 5.4% of all WS errors.

**Table 3:** String confusion matrix for wrong string (WS) errors.

	**G**	**D**	**A**	**E**	**Total**
G	–	6	2	5	13
D	7	–	36	8	51
A	1	37	–	15	53
E	2	2	8	–	12
Total	10	45	46	28	129

*Rows: correct strings; columns: played strings*.

Table 4 shows the indicated direction of string crossing (based on the fingering chosen by the performer) vs. the actual performed direction for the WS errors. Where the indicated and performed directions are the same, either the number of strings crossed is incorrect (e.g., moving up two strings when the passage calls for moving up one) or more than one note in a row was played on the wrong string.

**Table 4:** Indicated vs. performed direction of string crossing for wrong string (WS) errors, from the preceding note to the incorrect note.

	**Down**	**Same**	**Up**	**Total**
Down	17	12	12	41
Same	18	10	16	44
Up	10	19	15	44
Total	45	41	43	129

*Rows: indicated (correct) direction; column: performed direction*.

WS errors were almost equally likely to occur whether the correct action was to move up a string, down a string, or to remain on the same string. The last of these cases is notable, as it shows that performers spuriously played string crossings even where none were called for. By contrast, only 22 of 129 WS errors (17%) were caused by the bow moving in the opposite direction to what was indicated (i.e., moving up when it should be down, or vice-versa).

### 7.1. Discussion

The result of this investigation revealed that errors were performed with the reverse violin only, thus disproving our hypothesis that performances with small violin would also result in a number of double stops. The most common error was *wrong string*, i.e., the performer bowing the wrong string while placing the left hand finger on that same (incorrect) string. This type of errors demonstrates continued coordination between left and right hand even as the action is inappropriate for the layout of the instrument. By contrast, the less common *open string* errors may represent a dissociation between the left hand figure placement and choice of string to bow.

The confusion matrix of strings and the pattern of errors with respect to the direction of string change did not reveal any clear trends, showing that the bowing errors cannot be accounted for with a simple mirroring model where the performer moves in the opposite direction to what the passage calls for. It also emerged that players who produced more of one kind of error did not necessarily produce more of another, suggesting that each player encountered different challenges in playing the reverse instrument.

## 8. Bowing gestures

The data collected by the motion capture system was used to analyse the bowing gesture for the Bach *Courante* to bring quantitative evidence to the predictions and the corrections performed by the violinists. For each performance we computed the angle of the bow with respect to the body of the violin. There were gaps in the bow data, but the spatial difference between the points was marginal, so bow angle data was calculated using whichever points were available. The violin scroll, left and right markers were used to create a plane, and two bow points were used to calculate the normal and then the angle between them.

**Figure 8:**
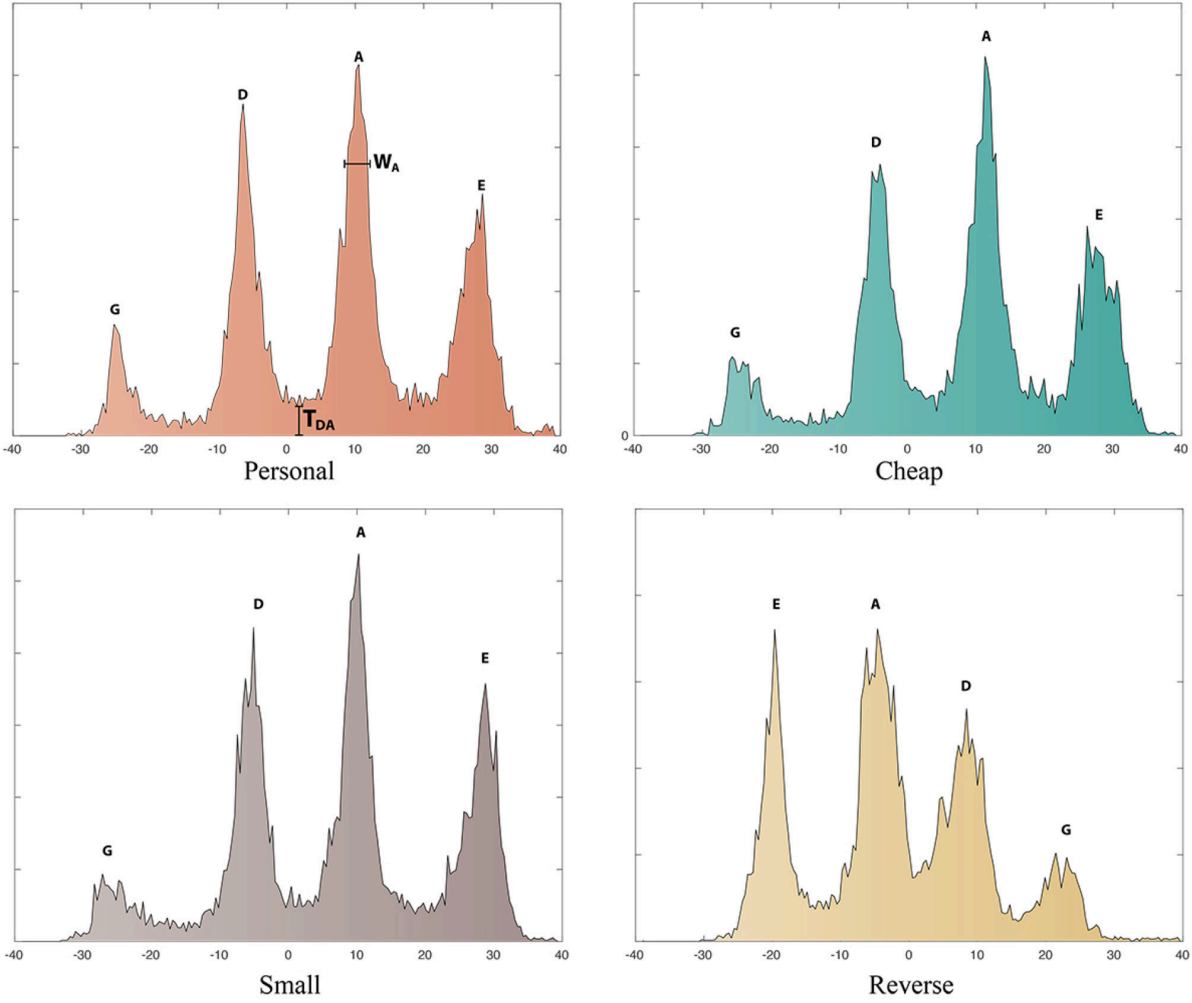
Histogram of the bow angles relative to a single performance played with the personal **(Top left)**, cheap **(Top right)**, small **(Bottom left)**, and reverse **(Bottom right)** violin by *P3*. G,D,A,E indicate the position of each string; *T*_*DA*_ indicates the trough between the strings D and A; *W*_*A*_ indicates the width of the peak relative to the A string.

The bow angle data was analyzed by computing the histograms of the angles for each performance (Figure 8 shows the computed histogram for the bow angles with the four instruments for one particular violinist, P3). This analysis allowed us to inspect the location and width of each peaks. The sharpness of the peaks indicates the accuracy/precision of string selection; we expected the frequency of the angles to be clustered around 4 peaks, one for each string. The width of the peaks might indicate the extent to which performers had to correct their initial predictions by varying the angle of the bow. Wider peaks would indicate more variance in the bow angle, where higher troughs between the peaks would indicate either significant numbers of errors or a larger number of string crossings. However, the width might also reflect deliberate (if unconscious) strategies to play closer to a neighboring string for convenience.

Our hypotheses would predict that the reverse instrument would have wider peaks and higher troughs than the other instruments. Questions also related to whether the small violin required greater precision, in which case the strings are closer together and peaks are narrower, and whether the other violins had peaks in different locations, which might be expected given different shapes of the bridges.

### 8.1. Results

**Figure 9:**
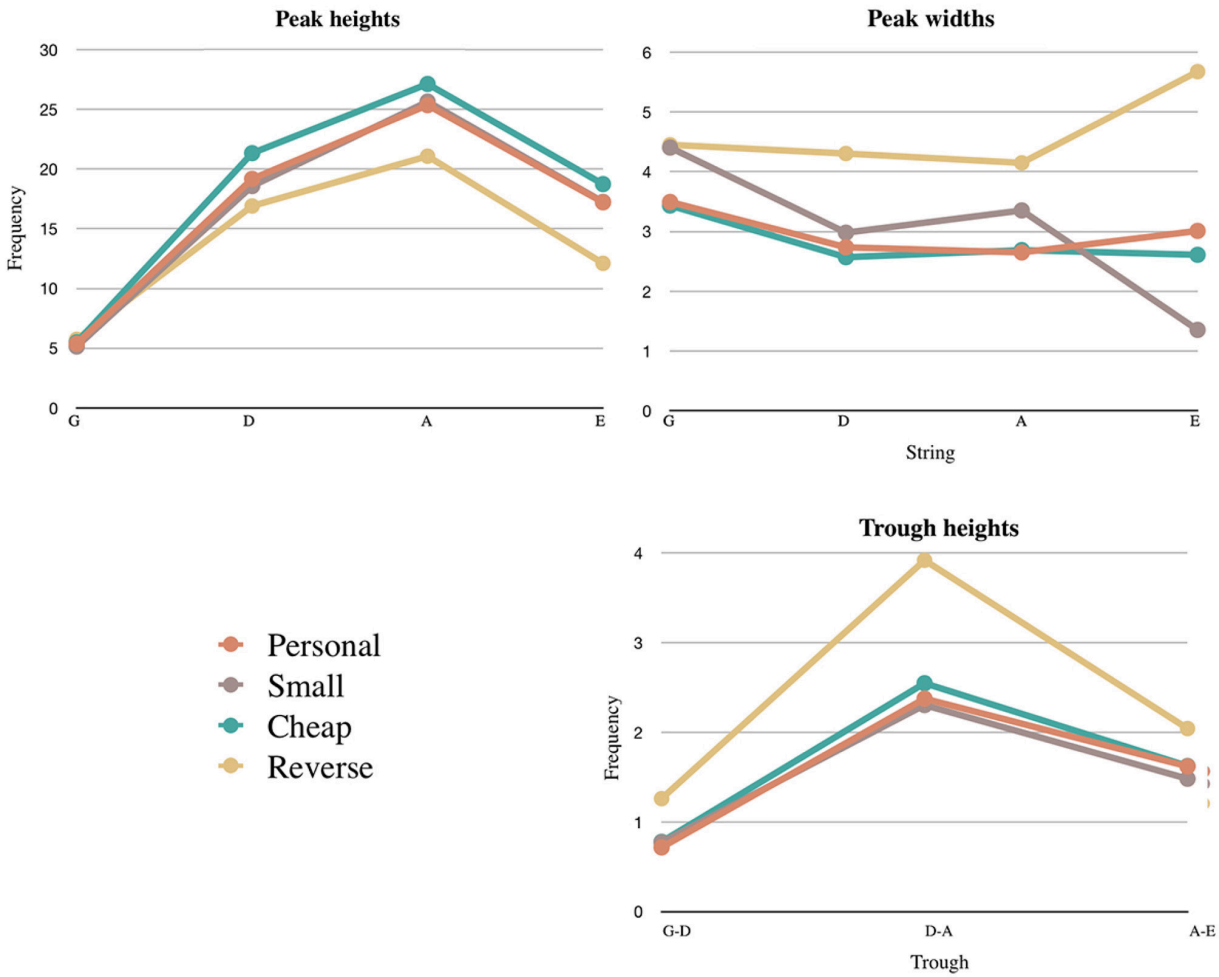
Means of the peak heights, widths, and troughs for each instrument. This data accounts for the reversing of the strings on the last violin.

Figure 9 shows the heights, the widths, and the troughs of the peak for each string in the four different conditions. It is visible that the peaks of all strings have similar behaviors, with the exception of the *A* and *E* strings, which are lower for the reverse instrument. The behavior of the peak widths appears less uniform; the central through is visibly higher for the reverse violin. This trough represents the space between the D and A strings, and indeed spuriously playing both of these strings as a double-stop was a common error for most violinists.

We tested the statistical significance of these observations. Two one-way within-subjects ANOVAs were conducted to test the influence of instrument on the width and height of the peaks on each string. The main effect of instrument was statistically significant on peak height (*F* = 8.070, *p* < 0.005, partial μ^2^ = 0.669) but not on peak width. Once this trend was proven, a *t*-test (two-tailed) was performed to compare the height of the peaks of the personal violin against the other three conditions. The peaks of the reverse violin were significantly lower than those of the personal violin (*t* = 3.071, *df* = 23, *p* < 0.025).

The height of the trough (*T*_*DA*_) were then compared with a two-tailed *t*-test: the trough for the reverse violin were significantly higher than those of the personal violin (*t* = −2.916, *df* = 17, *p* < 0.025). Finally, a two-way within-subjects ANOVA was conducted on the influence of instrument on the location of the strings. The main effect of instrument was not statistically significant [*F*_(1, 12)_ = 3.007, *p* = 0.072, μ^2^ = 0.429)], meaning that the position of the strings did not vary significantly across the different instruments.

#### 8.1.1. Discussion

We hypothesized that participants would need to perform several string crossings to correct bowing errors when playing the reverse instrument, a phenomenon that could be observed by assessing whether performances with the reverse violin had wider peaks and higher troughs than the other instruments. Our analysis revealed that this was not the case as the influence of instrument of peak widths was not significant. The lower heights of the peaks of the reverse violin might suggest that violinists struggled finding the correct angle of the bow, especially with respect to strings A and E (which, on the reverse violin were tuned as a D and G, respectively). We also hypothesized the small violin to have more narrow peaks due to the higher precision that is required. This hypothesis was not supported by our results.

## 9. Interview

### 9.1. Cheap Violin

When asked to comment on their experience with the cheap violin, some participants (P2, P3, P4, P5) reported issues they normally do not have with their violin: “*My attention went to achieve the pitches, and I could not think about shaping and structure*” (P2); “*It felt strange. I had to give up timing details*” (P5). The difficulties for P3 and P4 were connected to the different measurements of the fingerboard. P3 explained that such difficulties “*always happens when you change to an instrument, but maybe in particular when you have cheap instruments*.” By contrast, this problem did not seem to occur for two others performers (P1 and P7): “*I did not find this instrument more difficult to play than my own*” (P1).

### 9.2. Small Violin

The comments of participants suggest the idea that the small violin required them to adapt their playing. When discussing her experience with the small instrument, P6 commented: “*There is a tremendous amount of more adjustments that you need to make to be able to play at all. Huge adjustments on the left hand because the strings are so close together. It is difficult to play one string at a time. The intonation is very different, it's very hard to adjust to*.” Also, when playing the small violin, they had to dedicate more conscious attention to playing accurately. For instance, P3 reported: “*I needed to pay attention to stay really close with the hands, the distance between the fingers had not to be open. For the intonation. And then when I changed positions, it was a bit difficult to find the right place*.”

The morphological characteristics of the small violin make it difficult to be played by an adult: “*Since the distance is smaller, it makes it easy to make wrong notes. The tiny difference will make huge change*” [P1]; “*On a normal size violin you've got to be accurate on a tenth of millimeter, on that [the small instrument] you have to be accurate on hundredth of millimeter*” [P7]. P4, commenting on the issues she had with the small violin, said: “*shifting positions was difficult because the measurements are different. I was not accurate with that and also the articulation*.” Some players commented that they developed strategies for adjusting to the structural changes of the small violin. For instance, P2 said that she found it very difficult to shift position with the small violin; the strategy she adopted for improving this situation was to listen to a short glissando.

### 9.3. Reverse Violin

The experience of playing the reverse violin was particularly frustrating for all violinists insofar as they had to dedicate full conscious attention to adapting their playing to the different configuration of the violin. “*It was very tricky in the mind because you have to organize the movements. And to predict what you have to do to find that note, that you used to find there but is in another place*” [P3]. A direct consequence of the disruption of attention was the termination of the automated response. P0 commented: “*[the experience of playing the reverse violin] is like reading something with a heck a lot of accidentals. I can't just fall into my default, I have actually to parse it*.” She added: “*It was really hard to play something by memory just because I couldn't think of using the motor memory, and that's half of how I play a piece by memory. For the pieces I knew I had to overwrite my natural inclinations whereas for sight reading I was just parsing and thinking about the mechanical relationship between where it goes on this altered violin. I think my sight reading sounded better than my Bach*.” The unfamiliar instruments not only prevented musicians from relying on familiar automatization but also affected their very experience of playing insofar in that their flow was constantly disrupted. “*When playing this instrument [reverse] I have to concentrate on what the actual notes are, rather than just doing it. So, it breaks the flow too much. Which means that I can't do that music because I have to think what the actual notes are, what actually I would mechanically have to change*” [P0].

Several participants reflected that they tried to find workarounds to compensate for the disruption of the automated motor controls. P5, for instance, reported that while playing the Courante with the reverse violin, “*I tried to the best of my ability to keep whenever possible in the same string. Most of it was just A-E or two strings*.” When commenting on the same piece, P7 said that her fingering choices were mostly done to avoid leaping a gap of one or more strings. P6 offered a similar comment: “*My brain was trying very quickly to sort out some sort of easy way to grab on what I thought I know about playing on each string like which direction I was going with the bow*.” However, their musicianship did not suffice to overcome the difficulties as they struggled applying the automatic sensorimotor processes they developed over many years of training. P2 commented that “*I was thinking about where to put the finger, what should I do*.” P6 added: “*having to switch your brain when you have literally done it for 35 years is like being a beginner*.”

Those violinists who were asked to play the Bach piece as if they were playing a normal violin, i.e., without worrying about the sounding pitches, all reported that this condition was much easier. Using the words of P7, this condition was easier “*because it is automatic on what string you are on. The fingers go where they should, and the same the bow.”*. P4 reported that it was easier for her to “*ignore the non-musical bit and focus on the movements.”* P6 tried to elaborate on this aspect suggesting that “*I know where my fingers are supposed to be going down to make the right note even if I can't tell what these notes are. Because they are different from the notes I would expect so I am not hearing it except in my head at all. I am not pitching it to my head.”* Interestingly, similar comments about ignoring aspects of auditory feedback were provided by P0 about the experience of playing the small violin. She explained how she is normally aware of the pitch she is playing by means of the way it rings with the violin. With the small violin she said that “*I was completely not playing on in-tune strings. So I was trying to ignore it and just be like: well my fingers seem still.”*

## 10. Discussion

In this section we discuss a number of themes that we identified by integrating the comments collected from the performers with quantitative results.

### 10.1. Instrument Alterations Affect Transparency

The comments collected from the interview clearly point to a significant attentional demand while playing the altered violins, consistent with a loss of transparency. In particular when performing the reverse violin, participants could not rely on automated sensorimotor processes and were forced to consciously plan their actions. An explanation comes from the *adaptive mechanism* concept from Gentili et al. (2015): our participant's brain had to focus on the new “environment” while at the same time suppressing the automatic responses of existing pathways. These additional cognitive load resulted in a self-reported lack of expressivity. This result confirms previous the results of previous studies that offered the idea that musicians have to give away expressiveness when facing other cognitive load (for instance carrying out a secondary task) (Çorlu et al., 2015).

The quantitative data that we collected offer a detailed understanding about the disruption of sensorimotor mechanisms that resulted in a loss of transparency. The initial prediction was significantly inaccurate in both altered conditions; violinists struggled in particular with the reverse violin, suggesting that axis-inversion interferes with generalization of violin playing. The duration of the performances with the reverse violin were 2-4.8x longer than those in normal and scaled-down conditions, with halting and error-filled performances observed in the reverse condition. Significant bowing errors were observed only with the reverse violin, and at times it took more than one repeated attempt for performers to find the correct finger-string combination. The analysis of the bow angle also suggested that the initial prediction needed significantly more adjustment in the reverse condition. The height of the trough between D and A (the two central strings) suggests that they particularly tended to confuse the two strings or bow the double-stop between them. This observation is confirmed by the error analysis and by a comment from P7: “*I can't get the A and D strings the other way around. The G string and the E string are easier somehow to know in what string I am in.”*

These reflections offer information about the extent to which the ability to play an instrument generalizes to structural modification. Using the classification of generalization proposed by Krakauer et al. (2006), the generalization of our participants' ability *transferred* with the cheap violin, whereas an *interference* effect was obvious on the reverse violin. The small violin may have aspects of both transfer and interference, in that conscious attention and adjustment were required, but the basic fluency of the performance remained intact. These results resonate with findings in other domains which suggest that expertise transfer is hard to achieve. For instance, speech learning is highly sensitive to changes of context and does not transfer even when utterances involve very similar movements (Tremblay et al., 2008).

### 10.2. Prediction Mismatch Creates Confusion

The effect of musicianship and physical familiarity resulted in another notable observation. For those violinists who were asked to play the Bach piece as if they were playing a normal violin, their ability to anticipate movements did not seem disrupted even though auditory feedback on pitch was scrambled. The sequence of pitches produced by the reverse violin played as if it was normal might be similar to the random-pitch condition in Pfordresher (2005), suggesting that this alteration of auditory feedback would not be significantly disruptive.

One of the most unexpected results came from the analysis of the bowing errors, which indicated that many errors were made that were not related to axis inversion. Specifically, 41 errors occurred when the music did not actually demand a string change. One possible explanation is that the automatic response of the player is to associate a particular note with a particular place on the instrument, rather than thinking only relative to their current position. Another explanation is that the mismatch between their predictions and their current experience created a sort of confusion, which inhibited the access to their internal representation. A few comments collected from one violinist, P0, support this theory. “*For most of these pieces I have an unconscious reaction because I've memorized them. And I can't actually tell you what I am doing, which means if I mess up while I am playing I am usually completely screwed up because I don't know what I was doing. My fingers and my physical memory has gone off. So, playing this I had to concentrate on what the actual notes are, rather than just doing it.”* She added: “*Playing the Bach, where I actually know where the melody is quite well in my head was actually a detriment because thinking about what the next note is…I have a very strong relation mentally with where my fingers should go, and my bow should go, which is now broken.”*

Another possible explanation is offered by the concept of *coarticulation* (Godøy et al., 2010). This concept postulates the idea that the same note (or other musical outputs) can be produced differently depending on what note (or musical output) precedes or follows it. That is a consequence of motor learning that allows the musician to incorporate the actions prior to and following the current one into the ongoing movement recovery and preparation of the current action. These unexpected errors seen in the reverse instrument might reflect issues in coarticulation as a result of disruption to the surrounding notes of the sequence.

### 10.3. Adaptation Is Personal

The quantitative analyses suggested that the robustness of existing sensorimotor skills to changes in physical form seem to be highly personal. Even though all 7 players were expert musicians, some of them were much faster and more precise at adjusting their predictions. The analysis of intonation showed that 2 performers were able to correct their initial predictions with the altered instruments as accurately as with the non-altered instruments. This was not the case for the other performers: some struggled more with the reverse violin, some struggled more with the small one, and others equally struggled with the two altered violins. The fluency of their performances also greatly varied. Some violinists kept halting and committing errors with the reverse violin, whereas the performance of others (in particular P2) looked nearly fluent, if still slow. The ratio between the time it took to perform the Bach piece in the reverse vs. personal violin ranged from 2 to almost 5. The analysis of bow error further revealed that each player faced different challenges in playing the reverse instrument: the number and type of bowing errors greatly varied amongst performers. The capability to adapt to micro-structural changes also emerged in the interviews that followed the performances with the cheap violin. Whereas most performers did not report any particular issue, two of them noted that their had to dedicate more attention to fingering thus having less “cognitive space” for expressivity and musicality.

### 10.4. Future Directions

The aim of our study was to understand how changes to instrument structure after the instrument's functional transparency to the performer. Earlier in this paper, we speculate on the possible existence of a *transparency bandwidth*. Our experimental design, which considers one example each in two classes of alteration (scaling and axis-inversion), does not allow for pinpointing a specific threshold beyond which transparency disappears, though it does show the relative sensitivity of various technical aspects of playing to these two types of alteration. Future studies seeking to identify a transparency bandwidth might include several gradations in instrument design, particularly in size. It remains an open question whether intermediate designs between normal and axis-inverted conditions can exist.

Future studies could also investigate the specific causes of disrupted fluency on the reverse violin. A possible experiment to separate feedforward and feedback mechanisms would be to renotate a piece to be played “as found” on a reverse violin, such that playing the written notation results in the pitches of the original music being sounded. Notation for *scordatura* string instruments will often indicate to the player what pitch would sound if the instrument were normally tuned. Open questions under this condition include the fluency of performance, the presence of bowing errors, and whether the performer would be able to adjust their intonation as quickly and accurately as on their own instrument.

## 11. Conclusion

Related studies suggest that performance fluency transfers to new instruments after a period of re-adjustment (Bijsterveld and Schulp, 2004), which we suggested may be a consequence of a process of updating the forward and inverse internal models (Wolpert et al., 2001). We aimed to investigate the extent to which an instrument can be structurally altered before its transparency breaks down. To do so, we examined robustness of existing sensorimotor skills to changes in physical form by operating a number of instrument alterations, inspired by related work on learning generalization (Bock, 1992) and test how they impacted musicians' predictive mechanisms.

Our results showed that the sensorimotor processes that are necessary for instrument transparency were disrupted in case of reverse violin (axis-inversion mapping) and suboptimal performances were observed in the case of scaling. The implication is that the prediction mechanism, which is acquired throughout years of playing and that are necessary for proficient performances (Zatorre et al., 2007; Novembre and Keller, 2014), is a function of the construction of the instrument. Specifically, sensorimotor predictions seemed to depend on string orientation and on the scale of the instrument. Even small differences in instrument construction were noticed by some performers, who perceived small differences in the lengths of the fingerboard of the personal and cheap violins as something that was difficult to deal with.

Our findings open a number of questions that can be addressed by future studies. First, the present study demonstrates that the prediction mechanism depends on the construction of the instrument but does not precisely identify thresholds above which the transparency effect is disrupted. For instance, what is level of scaling above which sensorimotor mechanisms do not adapt (in our study the scaling factor was 0.77)? What musical material would the violinists would be able to play skilfully on the reverse violin? For example, could they play a single-string exercise accurately, given no anticipated string-crossing disruptions? Our results suggest that the capability of sensorimotor predictions to adapt seems to be highly personal. However, we are not able to exactly pinpoint what factors contribute to such variability. We invite future studies to further explore these issues with carefully designed, and perhaps longitudinal, experiments.

The findings of this study on the musician's ability to adapt to instrument reconfigurations extends to other research areas, and in particular to that of musical instrument design. The community of designers of new musical instruments have already noted the importance of repurposing existing skills when designing for expert musicians (Cook, 2001) while admitting that it remains unclear how to do this (Jordà and Mealla, 2014). When the instrument has different size, we observed, the performance fluency is not particularly affected, but axis-inversion adaptations disrupt it. However, in both conditions performers seemed to have some real problems with intonation. Furthermore, our result about the performers' different ability to adapt to structural changes suggests that the design process of new or augmented instruments should account for this diversity by including the performer in the design process. Finally, our participants demonstrated an aptitude to find alternative ways to perform when the traditional way of playing it was obstructed, a relevant aspect to be considered when designing new interaction with a musical instrument while maintaining existing skills.

## Data Availability Statement

The datasets generated for this study can be found in the website of the lab http://instrumentslab.org/data/GeneratedDataViolinStudy.zip.

## Author Contributions

FM and AM designed the study and wrote the paper. FM collected mocap data, conducted the *intonation* analysis. AM conducted the experimental study, performed the *bowing error* analysis. JA processed the motion capture data.

### Conflict of Interest Statement

The authors declare that the research was conducted in the absence of any commercial or financial relationships that could be construed as a potential conflict of interest.

## Footnotes

^1^We also collected information about specific expertise with playing unconventional violins or techniques, including *scordatura* (P0, P3 and P7 have limited experience) and whether they use small violins to teach children (P2, P4, P5, and P7 occasionally do).

^2^Before the main study we conducted a pilot with one experienced violinist from our laboratory (female, 32 years playing experience). The results of her performance are not included in the numerical analyses because of changes to the experiment made in response the pilot. However, we included some of her interview comments in the Discussion section (she will be referred to as P0).

^3^This task was given to the last four performers only, as it was a comment that emerged with P3.

^4^http://instrumentslab.org/data/GeneratedDataViolinStudy.zip

^5^http://www.sonicvisualiser.org, Cannam et al. (2010)

^6^The cent is a logarithmic unit of measure used for musical intervals.

^7^We considered *wrong* all the notes whose error in cents was higher than 50.

^8^Where specific strings are referred to in this section, the names refer to the sounding pitch of the string. For example, G refers to the rightmost string on the reverse violin. Similarly, the correct string is that string which produces the correct pitch, not the one that would be in the expected position on a normal violin.
